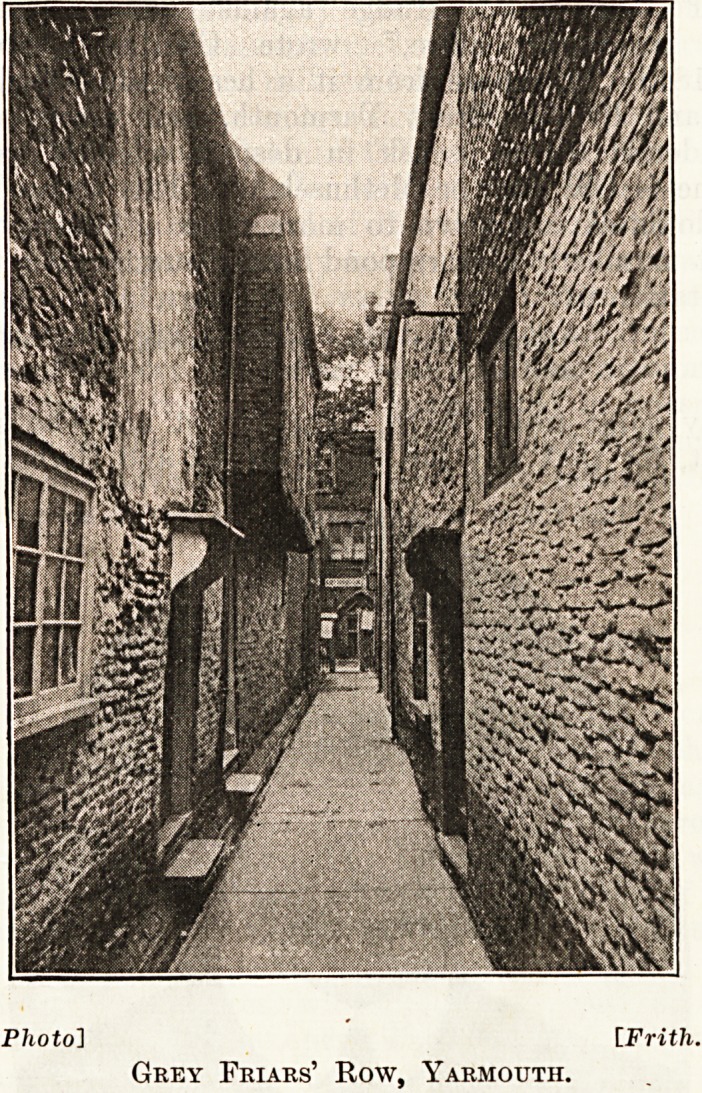# The Public Health: Interviews with Local Authorities—Yarmouth

**Published:** 1923-08

**Authors:** 


### THE PUBLIC HEALTH.

### INTERVIEWS WITH LOCAL AUTHORITIES.

### XI.—GREAT YARMOUTH.

"IF you bear a grudge against any particular insurance office," wrote Charles Dickens in 1849, "purchase from it a heavy life annuity, go and live at Great Yarmouth, and draw your dividends till they ask in despair whether your name is Old Parr or Methuselah." This testimony is doubtless unknown to more than a few of the vast numbers who respond every summer to the invitation to:

> "Come unto these yellow sands,
> And then take hands."

They come from North, South, West and—no, not East, for that way lies the open sea and Germany.

Councillor P. R. Hill, Chairman of the Great Yarmouth Health Committee.

And in the memorable years 1914-1918 they ceased to come from the North and the South and the West.

#### Where the Old are Young.

Councillor P. R. Hill, J.P., the Chairman of the Public Health Committee, and Dr. A. N. Stevens, the Medical Officer of Health, very courteously accorded us an interview on the subject of the health of this famous East Coast health resort. Our talk took place in the office of the Medical Officer in the Town Hall, an attractive, well-proportioned building which stands on a quay which for well over a mile runs alongside the River Yare, and is said to rank among the finest and most extensive in Europe. In view of Charles Dickens's picturesque statement, we were interested at the outset to learn that the death-rate (10.57) does in fact show a very high proportion of deaths among people who live to a ripe old age. No fewer than 43 per cent. of the deaths of Yarmouth residents occurred over the age of 65 years, and 25 per cent. over the age of 75 years. When, added to this, we state that the infantile death-rate is 69 per 1,000, compared with an average for the country generally of 83, Yarmouth can indeed claim to be a health resort. The figures are for 1921.

#### Two Invasions a Year.

It is refreshing to turn for a moment from the dark housing problems of the industrial towns, which overshadow all the health activities of the responsible authorities, to an area where not only is the normal population of 60,000 housed, but a double invasion each year is adequately provided for. There is the invasion of the visitors in the short summer season, which swells the population enormously; in the possible effect of this on the housing question the authorities see nothing amiss. They possibly remember that Nelson, to whose memory a monument stands on the South Denes, was none the less a good Admiral because he had a blind eye. The second, and lesser invasion, that of the Scottish fishing girls during the fishing season in October and November, is no longer a question of congestion in the neighbourhood of the fishing wharf because motor transport has come to the assistance of the merchants and the boat-owners, and the girls are comfortably accommodated in other parts of the town.

#### The "Rows."

Have you heard of Kitty Witches' Row or of Grey Friars' Row? We visited the Rows with these intriguing names and some of the 143 others which make so fascinating a feature of the general life of Yarmouth, although they doubtless escape the notice of many of the multitudinous merry-makers for whose entertainment the Corporation provide on that wonderful stretch of sea front from Caister to Gorleston—and, incidentally, reap a rich revenue in the process. We inquired whether these 145 picturesque rows were a source of anxiety to the Health Authority, because of their narrowness and the large number of houses to the acre. It was of interest to learn that, though there are many houses to the acre, there is relatively a small number of persons to each house; that the Rows all run at right angles to the sea and receive their full share of the easterly breezes, sweeping through them and keeping them sweet; and finally that the dwellings are so rapidly undergoing conversion into stores and warehouses that any question of overcrowding is rapidly solving itself.

#### The Homes of the People.

As to the homes of the people of Yarmouth generally, let the work of the two lady health visitors speak for itself. During the year 1921, in connection with the care of the children, 1,698 homes were visited. These were classified as follows:—Excellent, 69 per cent.; good, 22.6 per cent.; fair, 7.2 per cent.; bad, 1.2 per cent. General immunity from enteric diseases was noted here, as elsewhere, and, in connection with the work of inspection of vessels—of which there were 576 from foreign ports (please note that 117 of these were of German nationality) and 1,700 " Coastwise " arriving in the port during the year—it is worthy of note that sickness among the crews was negligible during the year 1921, two sailors only being removed to the isolation hospital with suspicious throats ; one was found to have diphtheria, the other not. Sixty-six canal boats were inspected, and all, without exception, were found to be very clean and well kept. There was no single case of sickness on board a canal boat during the year.

### The Winter Charm of Yarmouth.

In the month of August, in which these notes appear, we commend to those whose holidays do not fall within this popular month the delights of Yarmouth out of what is called the Season. There is no real reason why Yarmouth should not have a winter season. It has a drier and warmer climate than places farther from the coast. Its daily mean record of bright sunshine for 1921 was 5.38—a scrupulously kept record, too. And if the east wind is stiff at times, it is undoubtedly invigorating (the backbone of the Englishman, Charles Kingsley called it). And as you will have less inclination to

Photo] [Frith.

Kitty Witches' Row, Yarmouth.

" laze " upon the piers or sands, so you will have all the more time to get to know this really interesting town, to see for yourself the wonderful activities of the harbour in the herring season, to learn at first hand what a " cran " of herring is, and to see on a busy day on the wharf what a few thousand crans look like all at once ; to see some of the Elizabethan residences on the quay and study some of the contents of the old Tolhouse Museum ; to chaffer in the fine market-place on market days, and visit the grand old church of St. Nicholas ; to wander in the Rows and along the quay and amongst the old red-

Photo] [Frith.

Grey Friars' Row, Yarmouth.

roofed buildings and sheds, along the southern side of the river, seen so effectively from Gorleston Jetty at the harbour mouth.

### From Nelson to Beatty.

There is much of literary interest in a town which contains the site of Peggotty's Hut and other spots dear to the readers of " David Copperfield," and of Defoe as well as Dickens. And you may get impregnated with the salt and the spirit of the air in which were bred the fishermen who played so great a part in the Great War—the air of a town which is honoured by, as it does honour to, the name of Earl Beatty on the same roll of freemen as that of Lord Nelson. It is the business of Mr. Hill and the Health Committee of which he is Chairman, and of Dr. Stevens and his staff, to keep this interesting town of Yarmouth sanitary and clean for those who dwell there, as we have seen, to a ripe old age, and for those who visit the town and will visit it, let us hope, in increasing numbers in a new season of the year. Very worthily do these health authorities discharge their task.

---

### St. David's Hospital, Bangor.

The negotiations between the Bangor Guardians and the Carnarvonshire and Anglesey Infirmary, Bangor, for the purchase or lease of the board's St. David's Hospital have been abandoned by both parties. The Guardians propose to occupy St. David's Hospital, and to transfer to it patients from the workhouse infirmary. Thus a long continued state of uncertainty has at last been brought to an end.

## Figures and Tables

**Figure f1:**
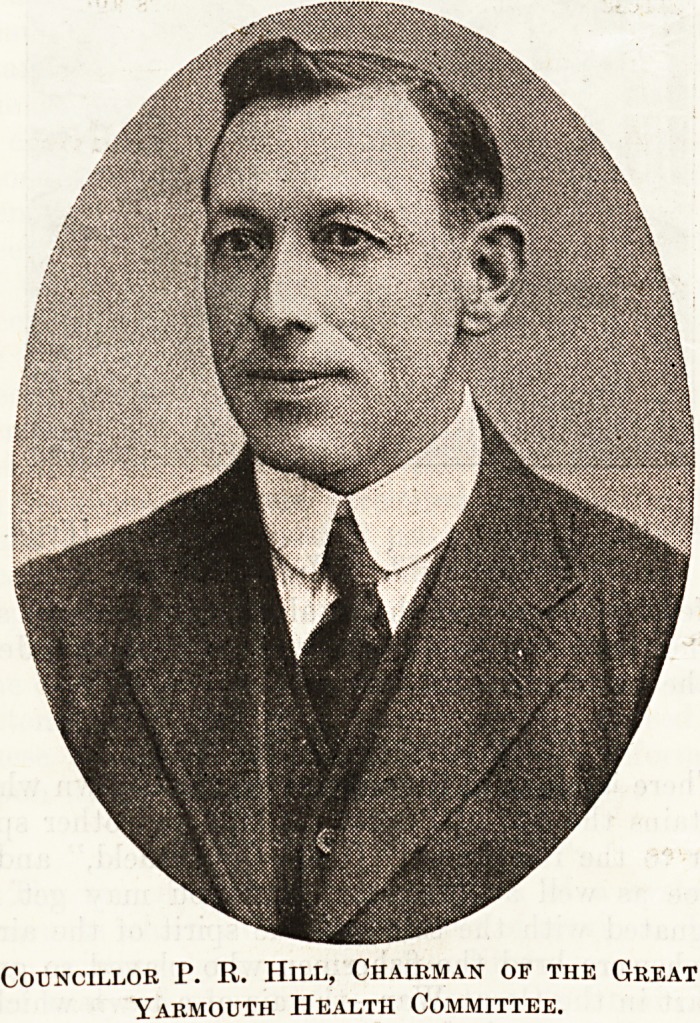


**Figure f2:**
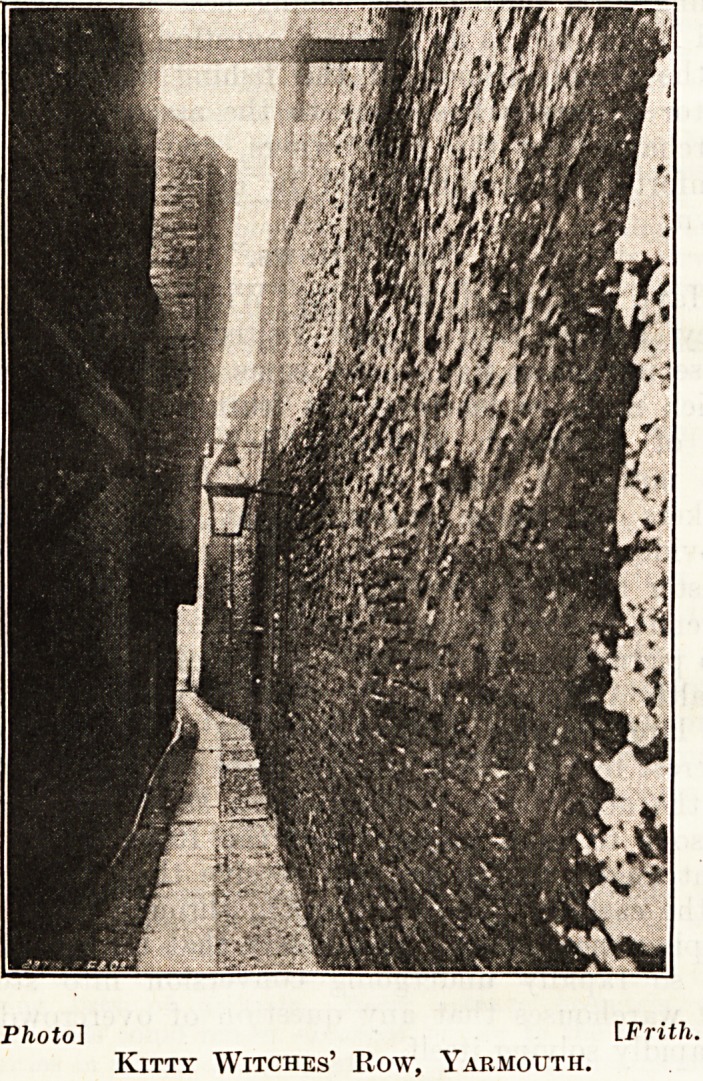


**Figure f3:**